# Gas nanosensors for health and safety applications in mining

**DOI:** 10.1039/d3na00507k

**Published:** 2023-10-24

**Authors:** Mahroo Baharfar, Jiancheng Lin, Mohamed Kilani, Liang Zhao, Qing Zhang, Guangzhao Mao

**Affiliations:** a School of Chemical Engineering, University of New South Wales (UNSW Sydney) Sydney New South Wales 2052 Australia guangzhao.mao@unsw.edu.au m.baharfar@unsw.edu.au; b Azure Mining Technology Pty Ltd Sydney New South Wales 2067 Australia; c CCTEG Changzhou Research Institute Changzhou 213015 China

## Abstract

The ever-increasing demand for accurate, miniaturized, and cost-effective gas sensing systems has eclipsed basic research across many disciplines. Along with the rapid progress in nanotechnology, the latest development in gas sensing technology is dominated by the incorporation of nanomaterials with different properties and structures. Such nanomaterials provide a variety of sensing interfaces operating on different principles ranging from chemiresistive and electrochemical to optical modules. Compared to thick film and bulk structures currently used for gas sensing, nanomaterials are advantageous in terms of surface-to-volume ratio, response time, and power consumption. However, designing nanostructured gas sensors for the marketplace requires understanding of key mechanisms in detecting certain gaseous analytes. Herein, we provide an overview of different sensing modules and nanomaterials under development for sensing critical gases in the mining industry, specifically for health and safety monitoring of mining workers. The interactions between target gas molecules and the sensing interface and strategies to tailor the gas sensing interfacial properties are highlighted throughout the review. Finally, challenges of existing nanomaterial-based sensing systems, directions for future studies, and conclusions are discussed.

## Abstract 2

The role of nanomaterials in advancing gas sensing systems for health and safety monitoring in mines.

## Graphical abstract

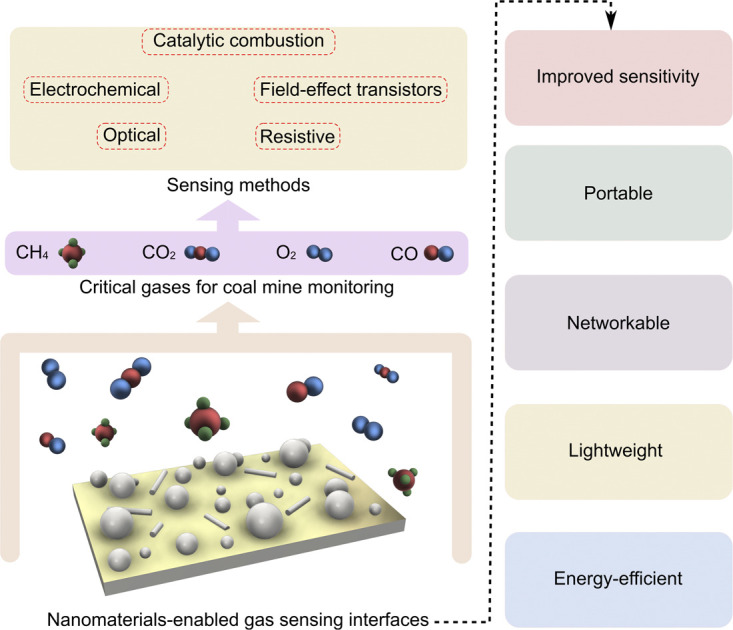

## 1. Introduction

Gas sensors are an integral part of health and safety monitoring in many industrial sectors ranging from healthcare to manufacturing and defense.^1^ The working environments in underground mines are known for their complexity and harshness with some hazards arising directly from various combinations of gaseous chemicals.^2^ The dynamic and tough mining environments pose serious challenges in maintaining occupational health and safety (OHS) of the workers in mines. A fatality rate of 2.9 per 100 000 was reported for Australia's mines in 2019.^3^ This number represents the fourth highest rate among various industrial sectors.^3^ Sustainable growth of the mining sector, which accounts for AUD$105 billion revenue for coal mines in Australia from 2012 to 2022 alone,^4^ requires investment in OHS monitoring technology and infrastructure of underground mines.

Since the emergence and commercial usage of gas sensors, a variety of gas sensing modules, configurations, and sensor/analyte interfaces have been introduced and their development remains at a fast pace.^5^ Advanced placeable, wearable, and implantable sensor technologies will play an increasingly important role in the future as envisioned by various health and regulatory organizations, employers, and researchers.^6^

Given the critical role of the sensing element in gas sensor performance, many of the efforts in gas sensor development are concentrated on exploring new sensing or transducer materials, for example, the metal–organic frameworks (MOFs).^7^ Commercial gas sensors generally contain bulk or thick-film sensing materials with limitations on the size, weight, performance, and power consumption of the overall sensor device.^8^ Replacing traditional sensing materials with nanomaterials thus promises to significantly reduce the size, weight, and power consumption in the next generation of nanomaterial-enabled gas sensors. Gas sensor miniaturization will drive new consumer products in portable or wearable electronics, internet-of-things (IoTs), and multi-gas detection technology. Sensors with sensing elements at the nanometer scale, *i.e.*, nanosensors, promise improved response time, sensitivity, and limit of detection (LOD).^1,9,10^ The recent strides in the design and implementation of nanomaterials have propelled significant progress in nanosensor technology, owing to the emergence of diverse classes of nanostructured interfaces. Noteworthy examples include nanoarchitectonic materials,^11,12^ few-layered 2D structures,^13,14^ 1D semiconductors,^15,16^ silicon carbide,^17,18^ and magnetic systems,^19^ as well as mesoporous^20^ and nanoporous^21^ materials. These nanosensing interfaces have demonstrated remarkably enhanced performance.

To date, a variety of nanomaterials have been employed to construct gas-sensitive interfaces in different configurations and with specific characteristics depending on the type of signal readouts.^5,9,22–24^ Gas sensing performance of these nanosensors is optimized by the size and shape,^25^ chemical composition,^26^ and interfacial assembly^27^ of the nanomaterials.

Despite the development of a wide range of nanomaterials for gas sensing, our understanding of the underlying gas detection mechanisms of nanomaterials is incomplete and therefore warrants further research.

The present review aims to provide a comprehensive overview of the current understanding of the operational principles and detection mechanisms of different gas sensing systems, along with a discussion of their advantages and disadvantages for their potential applications in the mining industry. To that end, we aim to capture the essential chemical environments in underground mines as well as current gas sensor technologies deployed for mine health and safety monitoring and unmet gas sensing needs of the mining industry. The role of nanomaterials in advancing such gas sensors toward fulfilling the needs of the mining sector for health and safety monitoring is highlighted in selective case studies. The interactive pathways between the nanomaterials incorporated into different sensing interfaces and the target gaseous analytes are examined to bring forth directions for developing nanosensors specific to the mining sector. Finally, gaps in the literature and motifs for future developments are provided.

## 2. Sensing modules of nanomaterial-enabled gas sensors

Nanomaterials have been incorporated into gas sensing interfaces based on different sensing modules.^1,5^ Depending on the combinations of the nanosensor material type and the target gas molecules, different sensing technologies have been introduced.^28^ In this section, we provide a summary of common sensing modules and the related sensing mechanisms.

### 2.1. Resistive gas sensors

Resistive gas sensors are among the most well researched sensors.^29^ The operational principle of these sensors is based on a change in the electrical resistance of the sensing material upon exposure to the target gas molecules. The resistance change is induced by chemisorption or physisorption of gas molecules on the surface of the sensing material.^30,31^ Metal oxide semiconductors (MOSs) are the most common type of nanomaterials used in chemiresistive sensors owing to their unique electronic structures and high numbers of active sites.^29,32^ MOS-enabled nanosensors detect gases based on the adsorption and ionization of oxygen molecules in a typical temperature range of 200–400 °C.^33^ The oxygen ionization process leads to the removal of electrons from MOSs, forming an electron depletion layer (EDL) and a hole accumulation layer (HAL) in n-type and p-type semiconductors, respectively.^34^ Depending on the type of target gas molecules that interact with such electronic structures, electrons are either injected into MOSs (in the case of reducing gases) or removed from MOSs (in the case of oxidizing gases). For instance, in an n-type MOS, exposure to reducing gases leads to a decrease in the width of the EDL and a reduction in resistance; meanwhile, the interaction between oxidizing gas molecules and an n-type MOS increases the width of the EDL and resistance. A reduction in the concentration of charge carriers and increase in resistance are observed after the adsorption of reducing gas molecules on a p-type MOS. On the other hand, subjecting a p-type MOS to oxidizing gases results in an increased HAL width and a reduced resistance.^35–37^ MOS nanosensors are promising for gas detection in harsh environments such as underground mines. Their drawbacks include high operating temperature and limited lifetime of use due to surface poisoning.^38^

### 2.2. Gas sensors based on field-effect transistors (FETs)

A typical FET device consists of a sensing layer placed between a drain and a source terminal. An input voltage is applied to the sensing interface through a dielectric layer and a third gate terminal. In the context of gas sensing, the drain-source current is measured upon the interaction of the sensing layer with the target gas molecules at a given gate voltage.^39^ The flowing current can be modulated by adjusting the magnitude of the applied gate voltage, enabling a tuneable sensitivity for measuring different concentration levels of gas molecules.^40^ A wide variety of nanomaterials such as metallic^41^ and organic^42^ semiconductors, polymers,^43^ graphene,^44^ and carbon nanotubes (CNTs)^45^ have been used to construct FET nanosensors.^46^ FET-based gas sensors have several advantages over others including cost-effectiveness, low power consumption, and facile fabrication and miniaturization.^47^

### 2.3. Optical gas sensors

The operational mechanism of optical gas sensors is based on changes in the optical properties of the sensing nanomaterial after its exposure to gas species of interest. In this case, the sensing signal can be reflectivity, colorimetry, fluorescence, surface plasmon resonance (SPR), Raman scattering, absorbance, or refractive index.^48^ Optical gas sensors operate at room temperature and offer high chemical selectivity and fast response. However, challenges in integrating optics into electronic devices and their moderate sensitivity have restricted the real-world applications of optical gas sensors.^49^

### 2.4. Gas sensors based on quartz crystal microbalance (QCM)

Such sensors work based on the piezoelectric effect of quartz crystals where an interfacial mass adsorption perturbs the resonance frequency of the crystal. Therefore, the change in the resonance frequency upon exposure to target gases is recorded as the analytical signal.^50^ The sensing mechanism in QCM sensors is based on the adsorption and desorption of gaseous analytes, leading to a change in interfacial mass and resonance frequency. The integration of nanomaterials into QCM-based interfaces amplifies the available active sites for adsorption and desorption processes.^50^ To tailor gas-QCM interfacial interactions and improve the selectivity and sensitivity of the QCM gas sensors, a variety of functional coatings have been applied to QCM sensor surfaces.^51–53^ Compared to other types of sensors, QCM sensors are compatible with a wider range of nanomaterials. These sensors operate at room temperature and require low power for operation. However, improvements in their sensing stability, reproducibility, and handling are required to meet the needs of the market.^53^

### 2.5. Electrochemical gas sensors

Depending on the electrochemical activity of the target gas molecules, this type of gas sensor employs different electrochemical techniques for quantification, such as amperometry, potentiometry, and electrochemical impedance spectroscopy (EIS).^54^ In these sensors, gas molecules diffuse through a membrane into a solid or liquid electrolyte toward the surface of a working electrode (WE), where an electrical input is applied to track subsequent electrical output events. The output current, due to the redox reaction of a target gas at the WE, is recorded as the sensing signal. EIS is an ultrasensitive and a more universal electrochemical sensing module that responds to changes in the interfacial chemistry of the WE upon gas adsorption. Interactions between the dissolved gas species and the WE surface result in variations in the impedance component, which can be used as the sensing signal.^55^ While electrochemical gas sensors offer high selectivity and sensitivity, they suffer from temperature sensitivity and leakage of liquid electrolytes. To enhance the performance and durability of such sensors, nanomaterials have been exploited to modify the WE surface for increasing the electrochemically active interface.^56^ Nanostructures have also been integrated to such systems as solid electrolytes to improve their durability and robustness.^57^

**Fig. 1:**
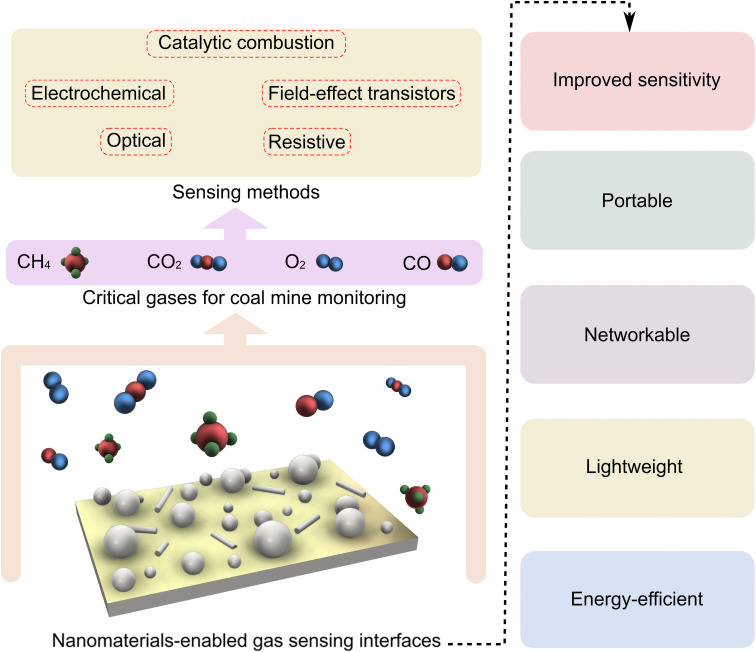
Schematic representation of a typical nanosensor interface, the gases of concern in coal mines, the common sensing methods utilized for mine monitoring, and the advantages of nanosensing interfaces. Nanoparticles and nanowires (grey objects) are predominantly used in nanosensor interfaces.

Apart from the sensing modules discussed above, there are other types of gas sensors such as surface acoustic wave (SAW),^58^ catalytic combustion,^59^ and thermal conductivity^60^ gas sensors. In brief, in SAW sensors, the properties (*i.e.*, amplitude or velocity) of an acoustic wave propagating through a material, are tracked upon the sensor interactions with the gas molecules.^58^ Catalytic combustion-based gas sensors use a sensing element with catalytic activity dispersed within a supporting matrix. Such sensors operate at an elevated temperature where the combustion of flammable gases, such as methane and hydrogen, further increases the temperature, resulting in a change in the sensor's resistance.^59^ Thermal conductivity gas sensors work based on measuring heat loss (change in resistance) upon the adsorption of gases with a lower thermal conductivity than air. The measurements are accomplished using a Wheatstone bridge circuit against a flow of a reference gas on a second sensing element.^60^Fig. 1 outlines the major sensing methods used to detect critical gas molecules relevant to mining safety, as well as the contribution of nanomaterials in enhancing the performance and applicability of these sensors.

## 3. Gas sensing needs in underground mines

**Fig. 2:**
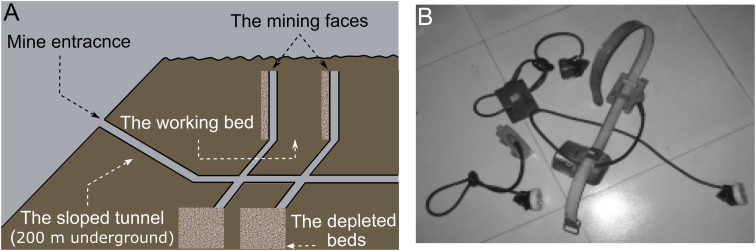
(A) Illustration of a typical underground coal mine. (B) Typical devices carried by miners. Reprinted with permission from ref. 61.

As shown in Fig. 2A, it is challenging to ensure health and safety of mining environments because of the size and shape of the underground roadways, typically with lengths of tens of kilometers and widths of several meters. Ideally many environmental factors, including the amount of gas, water, and dust, should be monitored continuously at many places throughout the tunnel, which demands high sampling density and number of sensor devices. Current mine environmental monitoring is typically conducted in a sparse and manual way due to the lack of advanced, reliable, and economical sensing techniques.^61^

The two major underground coal mining methods are the room-and-pillar (also referred to as the Bord-and-Pillar) method and the longwall method.^62^ In room-and-pillar mining, the coal is continuously cut and loaded onto a face transport vehicle (*e.g.*, a shuttle car) by a miner. In longwall mining, a longwall shearer does the same job, cutting and loading the coal onto a face conveyor on which it rides. The more recent longwall mining technology accounts for one third of all underground coal production. It is a continuous process using a rotating shear on the mining machine to cut into a block of coal. The coal is then removed by a conveyor from the mine. Desirable requirements relevant to the sensor technology include (1) remote management of the entire monitoring system including communication and routing mechanisms under all conditions and (2) *in situ* interactions with stationary sensors deployed on the walls, poles, and floors as well as mobile sensors integrated into devices carried by the miners (Fig. 2B).

The application of gas detection technology is significantly influenced by the challenging operating environment of gas sensors in underground coal mines. This environment is characterized by several factors, including wide fluctuations in atmospheric pressure, large temperature and relative humidity variations, high concentrations of dust particles, and strong electromagnetic interference. Additionally, there are other factors such as coal-rock collapse, mechanical vibrations, and unexpected impacts, which have varying degrees of impact on the gas detection devices. The production safety of underground coal mines is mainly dependent on the environmental conditions of the mines. The monitoring and maintenance system using traditional wire communication suffers from many shortcomings including high construction cost, damage of communication cables, high fault rate, inconvenient system maintenance, and others.^63^ As a result, a wireless sensor network (WSN) has emerged as an essential technology for continuous monitoring of the workplace environment in underground coal mines.^64^ Wireless operations impose strict requirements on power consumption of sensor nodes.^65,66^ A low degree of informationization and regular calibration requirements are the other main limitations in current coal production safety technologies.^67^ Therefore, it is of great significance to develop low-cost, low power consumption, and maintenance-free gas sensors based on new technology to detect various poisonous and inflammable gases.

The gases of relevance for coal mine explosion or fire are methane (CH_4_), carbon dioxide (CO_2_), carbon monoxide (CO), and oxygen (O_2_).^68^ The gaseous environments more relevant to iron and other metal mines can be found elsewhere in the literature.^69^ CH_4_, acetylene, hydrogen, and higher hydrocarbons are considered nontoxic but explosive. CO_2_, radon, and its daughter products are toxic. CO, sulfur dioxide, nitrogen oxides, and hydrogen sulfide (H_2_S) are acutely poisonous. Other impurities of concern are coal dust and water vapor.^66^

Underground fires can be caused by open flames, spontaneous combustion of coal, electricity, friction from cutting and drilling, welding, blasting, explosion, *etc.*^66,70^ Spontaneous combustion of coal is the main cause of fire in underground coal mines and ideally should be continuously monitored. Commonly used gas ratios and indices extracted from gas monitoring data to predict spontaneous combustion of coal are Graham's ratio, Young's ratio, and the oxides of carbon ratio, and the C/H ratio. Graham's ratio is the most widely used metric and is given by the ratio of CO produced to the oxygen consumed (ΔO_2_) in the process of spontaneous combustion. Graham's ratio = (100 × CO)/ΔO_2_. Young's ratio is given by the ratio of CO_2_ produced to O_2_ consumed. Young's ratio = (100 × CO_2_)/ΔO_2_. An increase in Young's ratio and decrease in Graham's ratio as a result of CO burning indicates the progress of fire from smouldering to open flame. The oxides of carbon ratio is defined as the ratio of the difference in the final and initial concentrations of CO and CO_2_. CO/CO_2_ ratio = (final CO − initial CO)/(final CO_2_ − initial CO_2_). The advantage of using this ratio is that it is uninfluenced by the inflow of air, nitrogen, or CH_4_. This ratio is a more sensitive indicator of fire than Graham's ratio. The C/H ratio is used to predict the intensity of fire along with O_2_ deficiency. C/H ratio = 6(CO_2_ + CO + CH_4_ + 2C_2_H_4_)/2(ΔO_2_ − CO_2_ + C_2_H_4_ + CH_4_) + H_2_–CO. For more information on these and other fire gas indices and how they are used to predict underground coal fires, refer to the excellent review by Muduli *et al.*^66^ While smoke detectors are a mature technology, work by Gottuk *et al.* showed that combining conventional smoke detectors with CO sensors can reduce false alarms while increasing fire detection sensitivity.^71^

The underground coal mine explosions are caused either by ignition of CH_4_ or coal dust or a combination of them. The release of inflammable gases from coal, CH_4_ and other minor gases (firedamp) can cause explosions. Real-time monitoring of CH_4_ and O_2_ is therefore critical for the detection and prevention of underground explosions. When CH_4_ buildup in an underground coal mine reaches a certain concentration range, 5–15%, explosion can be initiated by the presence of a small heat source. The minimum concentration of CH_4_ (in air) of this explosive concentration range is termed the lower flammability limit (LFL) (or the lower explosive limit (LEL)). The maximum concentration of this range is called the upper flammability limit (UFL) (or the upper explosive limit (UEL)). When CH_4_ concentration falls below the LEL, the amount of CH_4_ becomes too low to ignite. Similarly, the amount of O_2_ becomes too low when the CH_4_ concentration reaches above the UEL and no ignition occurs.^72^ Kundu *et al.*^73^ reviewed and summarized the explosion concentration range at different temperatures and pressures as well as the influence of various obstacles and geometries on explosions in an underground mine.^73^ CO and O_2_ sensors at the inlet and outlet of a working panel together with temperature sensors placed at the pillar junctions will enable real-time monitoring of health and safety risks to miners. The difference between the ratio of CO and O_2_ concentration at the outlet and inlet signal is used to detect fires when the difference is above a preset threshold. This results in activation of the temperature sensor nodes to identify the exact fire position.^62^

In addition to the major consequences caused by combustion and explosion of flammable and oxidizing gases, overexposure to certain gases in the mining environment could result in adverse effects on the health and safety of miners.^66,74^ Therefore, real-time and selective monitoring of certain gases in complex mining environments will meet a critical demand to ensure safe work conditions for mine workers. In the following subsections, we provide an overview of different nanomaterial-based sensing modules under research and development for detecting critical mining gases.

## 4. Nanosensors for critical gases in the coal mines

Traditional mine sensors monitor parameters such as temperature, smoke particles, and color of the fire to provide early warnings but with limited accuracy. Advances in gas sensor technologies have enabled the research and development of real-time, low-cost, and networkable gas sensors for mine and fire safety.^75^

Commercial chemical sensors deployed for gas monitoring in mining environments are usually based on bulk or thick-film materials.^76^ Such bulky devices require high power consumption and often contain limited diffusive pathways for gas molecules to interact with the sensor. Replacing bulk materials with nanomaterials thus allows device miniaturization with significant reduction in weight and power consumption. Nanosensors provide a larger surface-to-volume ratio of the sensing interface than traditional sensors leading to improved gas-detection sensitivity. Sensor miniaturization enables the fabrication of multilayered assemblies and interfaces or nanosensor arrays with tailored chemistry for enhanced gas-detection selectivity for a single gas as well as gas mixtures. Moreover, nanosensors offer faster response times owing to the improved diffusion of gas molecules and larger interfacial surface area.^1,5^

Popular mining gas sensors include sensors based on thermal conductivity^60^ and catalytic combustion,^77^ tunable diode laser absorption spectroscopy (TDLAS) sensors,^2^ and non-dispersive infrared (NDIR),^78^ and electrochemical sensors.^55^ Optical spectroscopic sensors such as TDLAS and NDIR sensors require large and expensive equipment and are challenging to integrate into portable or wearable optoelectronics. Thermal conductivity and catalytic combustion sensors are widely used for CH_4_ detection, but their performance is limited by the bulk nature of the sensing interfaces. Incorporating nanomaterials thus offers a solution to address unmet needs in gas monitoring in mining operations. Electrochemical sensors are typically used to monitor O_2_ and CO,^55^ but their real-world applications are limited by short lifetime and electrolyte leakage. Nanomaterials comprising solid electrolytes and molecularly structured ionic liquids have emerged as promising candidates for improving electrochemical sensing systems.^79,80^Table 1 provides an overview of the sensors commonly utilized for mine monitoring and their advantages and disadvantages. It also outlines the benefits and limitations associated with the integration of nanomaterials in each sensor.

**Table 1:** Technologies for monitoring critical gases in coal mines and their advantages and shortcomings. Advantages and shortcomings of nanomaterial incorporation are highlighted for each type of sensor

Sensor	Application in coal mines	Advantages	Disadvantages	Nanomaterial incorporation advantages/shortcomings	Ref.
Catalytic combustion	CH_4_ sensing	High sensitivity and low cost	Poor selectivity, surface poisoning, and frequent calibration	Formation of dispersed catalytically active sites and lower working temperature/challenges in mixing nanostructures with a supporting material (γ-Al_2_O_3_)	77 and 81
Thermal conductivity	CH_4_ and H_2_ sensing	Fast response	Low sensitivity and selectivity	Improving the permeability of the sensing material (ceramic beads) and increasing adsorption sites/challenges in the introduction and dispersion of nanomaterials into the supporting material	60 and 82–85
Resistive MOS-based^a^	CH_4_ and CO sensing	Miniaturized configuration and low cost	Poor selectivity, sensitivity to temperature and humidity, and surface poisoning	Possibility of fabricating multilayered assemblies to enhance selectivity, sensitivity, and response time/lack of a reliable and scalable manufacturing technology for the nanointerface	38, 82 and 86
NDIR	CH_4_, CO, and CO_2_ sensing	Room-temperature operation, high specificity, and fast response	High cost, large size, challenges in integration of electronics and optics, and easily affected by humidity and temperature	N/A^b^	49, 78, 87 and 88
TDLAS	CH_4_, CO, and CO_2_ sensing	Room-temperature operation, high specificity, fast response, real-time measurement, and maintenance free	High cost, large size, and challenges in integration of electronics and optics	N/A	2, 49, 82, 89 and 90
Electrochemical	CO and O_2_ sensing	High selectivity and sensitivity	Electrolyte leakage, temperature sensitivity, and poor durability	Incorporation of nanostructured solid electrolytes to improve durability and increase electrochemically active surface area/challenges in manufacturing nanointerfaces with long-term stability	79, 82, 91 and 92

a This technology, yet to be implemented in coal mines, is included here based on its potential applicability.

b In NDIR and TDLS, gas molecules interact with electromagnetic waves rather than nanomaterials.

As discussed above, existing sensing technologies for monitoring critical gases in mining environments fall short of the requirements for efficient, sensitive, and real-time sensing. Nanotechnology holds the potential to address the limitations of current systems and enable the development of next generation nanosensors specifically designed for complex mining environments and operations. In the following sections, an overview of the nanomaterial-incorporated sensors under development for the mining sector is provided. The gas and sensor interaction mechanisms are described wherever relevant throughout the review. Strategies for boosting the performance of nanosensors are also discussed.

### 4.1. Methane sensors

#### Resistive sensors

Most methane sensors studied so far are based on MOS-type resistive sensors. Such methane sensors mainly exploit an n-type semiconductor sensing layer where the exposure to reducing methane molecules results in an increase in the concentration of charge carriers at the interface and a decrease in resistance.^82^ Among different n-type sensing elements, tin oxide (SnO_2_) is by far the most studied functional material for methane^93–98^ and several successful commercial methane sensors have been developed based on SnO_2_.^99^ Further improvements in the performance of SnO_2_-based methane sensors have been accomplished through making composites, forming heterojunctions, and structural doping.^100,101^

**Fig. 3:**
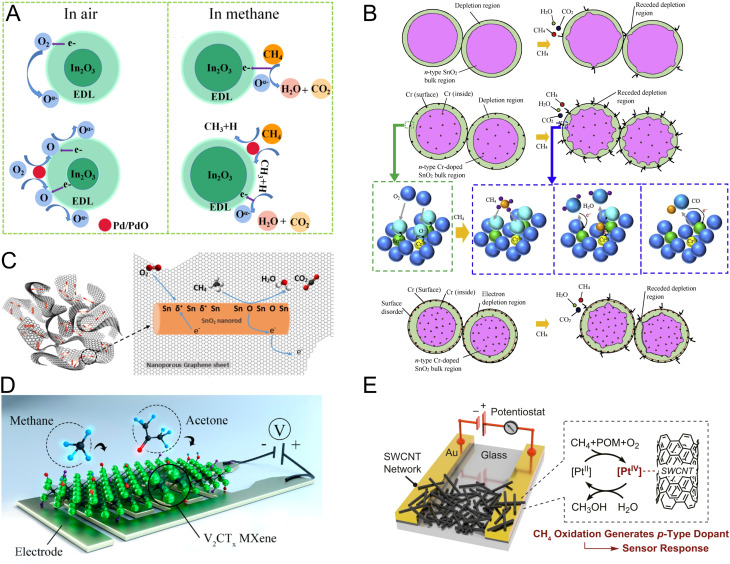
(A) Scheme of the chemical sensitization effect of noble metals incorporated into the sensing interface to improve the sensitivity of MOS sensors.^106^ Reproduced with permission from ref. 106 Copyright 2022 Elsevier. (B) Methane sensing mechanism of Cr-doped SnO_2_ structures based on successive methane oxidation and a decrease in the width of the depletion layer.^111^ Adapted with permission from ref. 111 Copyright 2019 Elsevier. (C) Charge carrier transport across dispersed SnO_2_ sites and nonporous graphene with a high surface area, enabling sensitive methane detection.^117^ Reprinted with permission from ref. 117 Copyright 2019 Elsevier. (D) Application of 2D vanadium carbide MXene for resistive methane sensing through the interaction of physisorbed methane with the surface functional groups of MXenes.^151^ Reproduced with permission from ref. 151 Copyright 2019 American Chemical Society. (E) Methane sensing achieved by the catalytic function and redox cycling of Pt sites in the SWCNTs/Pt-POM composite.^149^ Reproduced with permission from ref. 149 Copyright 2020 *Proceedings of the National Academy of Sciences*.

Generally, doping MOS structures, *i.e.*, SnO_2_ or indium oxide (In_2_O_3_), with noble metals such as platinum (Pt) and palladium (Pd) improves gas sensing performance through the formation of a wider EDL by enhancing interfacial oxygen chemisorption and ionization.^102^ This phenomenon is widely referred to as the “spill-over effect” or “chemical sensitization”.^103–105^ In addition to chemical sensitization, the presence of noble metal dopants in the MOS structure may lead to the formation of a “Schottky barrier” leading to electronic transmissions and charge separation at the metal/MOS interface, which is called “electron sensitization” (Fig. 3A).^106,107^

Due to known catalytic activity of Pd in the oxidation of hydrocarbons, Pd is one of the most researched dopants for MOS-based sensors.^108^ Depending on the operating conditions, in some cases oxidation of doped Pd to palladium oxide (PdO) at high temperatures is observed. This results in direct combustion of methane and reformation of non-oxidized Pd sites.^109^ In one example, Pd- and antimony (Sb)-doped-SnO_2_ interfaces exhibited excellent methane sensing performance in terms of sensitivity, response time, and reproducibility.^108^ The improvement in sensing properties of SnO_2_ upon doping was attributed to the catalytic effect of Pd on the dissociation of oxygen molecules and formation of oxidizing species as well as compensation of Sb^5+^ substitution in the SnO_2_ lattice leading to a reduction in the baseline resistance of the MOS sensor.^108^ In another study, pure SnO_2_ thin films (control) and films doped with different elements such as nickel (Ni), osmium (Os), Pd, and Pt were employed for methane sensing.^110^ The performance of the SnO_2_ thin film sensor was optimized by leveraging the results obtained from employing different dopants. Among the utilized dopants, Os appeared to improve methane sensing performance and reduce the working temperature of the sensor. Os was substituted into the SnO_2_ lattice as Os^3+^ with an unpaired d electron to catalyze first-step fragmentation of methane into hydrogen and CH_3_ radicals.^110^ In a similar study, Bunpang *et al.* prepared chromium (Cr)-doped SnO_2_ nanoparticles through substitutional incorporation of Cr (in the form of Cr^3+^) into the MOS lattice.^111^ Cr-doped SnO_2_ showed remarkable sensitivity (sensing response, which is defined by the ratio of initial sensor resistance to its resistance after exposure to methane, = 1268.6) and selectivity (evaluated against H_2_, C_2_H_2_, NO_2_, NO, N_2_O, CO, NH_3_, SO_2_, C_2_H_5_OH, C_3_H_6_O, and H_2_O) with a response time of 3.9 s at 350 °C working temperature and 1% methane. The improved gas sensing performance was explained by the surface area increase using doped MOS particles and generation of holes upon Cr incorporation, which leads to a decrease in electron concentration, increasing the width of the depletion layer. The gas sensing mechanism, shown in Fig. 3B, consists of several steps: (1) successive oxidation of methane to CO and CO_2_ and formation of reactive oxygen species (ROS) at the MOS interface, (2) desorption of ROS, (3) interfacial release of electrons, (4) formation of a receded depletion layer, and (5) an increase in the electrical conductivity.^111^

Another popular approach for improving the performance of gas sensing layers is based on the formation of composites and MOS-based heterojunctions.^112–115^ In a study by Vuong *et al.*, nickel oxide (Ni_2_O_3_)-decorated SnO_2_ composite films reduced the working temperature and enhanced methane sensing performance due to the synergistic effect of the composite material.^116^ In this case, a p–n heterojunction between Ni_2_O_3_ and SnO_2_ is formed. In addition, Ni_2_O_3_ depletes the electrons from an n-type MOS more than chemisorbed O_2_.

Other studies have employed carbonaceous nanomaterials for methane sensing.^103^ Kooti *et al.* employed a hybrid material consisting of SnO_2_ nanorods and nanoporous graphene.^117^ Their results show a substantial reduction in the operating temperature down to 150 °C and 600% increase in gas response compared to that of pure SnO_2_. This significant improvement was attributed to a higher surface-to-volume ratio and rapid charge carrier transport through SnO_2_ sites uniformly dispersed on conductive graphene (Fig. 3C).

Using similar strategies, other n-type MOS sensors based on In_2_O_3_,^106,118–123^ zinc oxide (ZnO),^124–132^ tungsten trioxide (WO_3_),^133,134^ iron borate (Fe_3_BO_6_),^135^ molybdenum disulfide (MoS_2_),^136^ and titanium oxide (TiO_2_)^137,138^ have been developed for methane sensing. In a study by Lu *et al.*, Pd–In_2_O_3_ was utilized for methane sensing and its cross-sensitivity to several interfering gases was observed.^139^ To maintain the selectivity for methane, they constructed a multilayer sensor consisting of a catalytic film on top of the sensing element. Catalytic filters of Pt–TiO_2_, Pt–cerium oxide (CeO_2_) and Pt–zirconium oxide (ZrO_2_), printed on the Pd–In_2_O_3_ layer, were effective in removing the background interference from CO, NO_2_, and ethanol. The basic sensing mechanism of a noble metal-doped MOS did not change after incorporating the catalytic filters.

There are some reports on p-type semiconductors for methane detection. Holes are the main charge carrier in the p-type materials. The chemisorption of O_2_ at high temperature removes the electrons from the conduction band of the semiconductor, thus increasing the hole charge carrier concentration and broadening the HAL. This results in a decrease in the resistance of the sensor. Exposure to a reducing gas such as methane releases the electrons back to the conduction band leading to the recombination of electrons and holes and an increase in the resistance of the sensor. So far, p-type interfaces based on tricobalt tetraoxide (Co_3_O_4_),^140^ lead sulfide (PbS),^141,142^ vanadium dioxide (VO_2_),^143^ iron oxide (Fe_2_O_3_),^144^ Co_3_O_4_/dicobalt tetraoxide (Co_2_O_4_),^145^ and copper(i) oxide (Cu_2_O)^146^ have been exploited for resistive methane sensing.

Other emerging nanomaterials applied to methane detection include MXenes,^147,148^ metallic complexes,^149^ and metal organic frameworks (MOFs).^150^ Lee *et al.* demonstrated room-temperature methane sensing by using 2D vanadium carbide MXene (Fig. 3D) reaching a limit of detection (LOD) of ∼9 ppm.^151^ Oxygen-containing surface functional groups of the MXene presumably provide necessary affinity for methane adsorption. In a study on metallic complexes for methane sensing, a composite of single-walled carbon nanotubes (SWCNTs) and Pt polyoxometalate (Pt-POM) showed selectivity and ppm-level sensitivity for methane detection.^149^ Sensing was carried out at room temperature and the sensitivity for methane was attributed to the catalytic activity of the composite and redox cycling of Pt (Fig. 3E).

#### Catalytic combustion sensors

For methane detection, a common sensor type employed in coal mines is the catalytic combustion type of methane sensors.^152,153^ The heat generated by methane combustion on a catalytic material is converted into an electrical signal in this type of sensor. A typical configuration consists of a catalyst embedded into an aluminum oxide (Al_2_O_3_) film mounted on a pellistor.^154^ Pt, Pd, Rh, and rare-earth perovskites are among the most commonly used catalysts in methane combustion sensors.^59,77,155,156^ In a study by Wang *et al.*, different catalytic systems of Pt–Pd/Al_2_O_3_, Pt–Pd/n-Al_2_O_3_, and Pt–Pd/n-Ce-Al_2_O_3_ were tested for combustion-based methane sensing.^81^ Their results show that doping the nanostructures with Ce presented anti-sulfur ability, lowered the reaction temperature and enhanced the catalytic activity owing to the redox properties of Ce.^81^

Methane combustion sensors are cost-effective, simple, easy-to-fabricate, and selective. However, they suffer from catalyst poisoning, saturation upon exposure to high concentration of gases, inaccuracy in small enthalpy changes, and high power consumption.^46^ To reduce the power consumption and improve the sensitivity of such methane sensors several strategies have been put forth, including application of a pulsed voltage to the bridge circuit,^157^ miniaturization,^158^ and exploitation of dual catalysts on hot and cold terminals.^155^

#### Electrochemical sensors

**Fig. 4:**
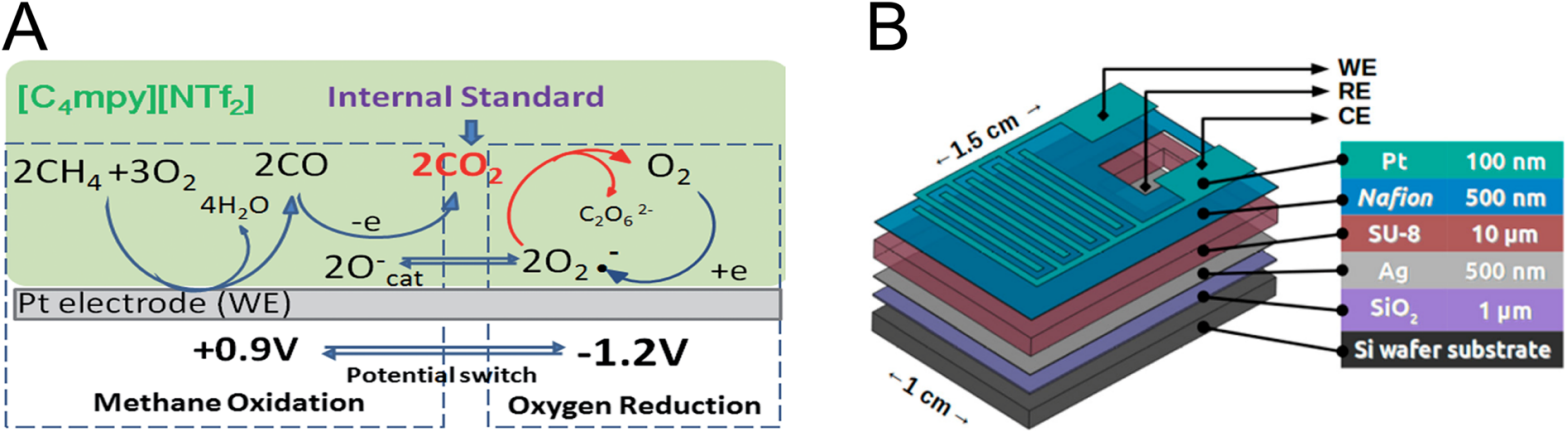
(A) Electrochemical methane sensing using IL-based electrolytes. CO_2_ generated by successive methane oxidation on the WE, and active oxygen species were used as an internal standard.^162^ Adapted with permission from ref. 162 Copyright 2014 Royal Society of Chemistry. (B) Scheme showing a multilayered electrochemical device based on solid-state electrolyte Nafion for methane sensing.^161^ Reprinted with permission from ref. 161 Copyright 2018 American Chemical Society.

Electrochemical sensing of methane is mainly accomplished through its oxidation reaction and resultant current changes based on gas concentration. Different electrode materials, catalysts, and electrolytes are being explored to improve the performance of methane electrochemical sensors. The earliest reports on methane electrochemical sensing were based on methane oxidation on a Pt electrode in liquid electrolytes.^159,160^ Due to the limited diffusion of gas molecules in liquid electrolytes as well as electrolyte leakage and evaporation, other types of electrolytes including ionic liquids (ILs)^80^ and solid-state electrolytes^161^ were explored. In the case of IL electrolytes, negligible vapor pressure, thermal stability, and a wide potential window improve the performance and lifetime of the sensors. In a study by Wang *et al.*, a pyrrolidinium-based IL electrolyte was used for simultaneous sensing of methane and oxygen. The sensing mechanism, shown in Fig. 4A, was based on the incomplete oxidation of methane to CO, followed by CO oxidation to CO_2_ by active oxygen species generated from the oxygen reduction reaction. The *in situ* produced CO_2_ was used as an internal standard enabling cross-validation and measurement error reduction.^162^

As a leakage-free and thermally stable class of electrolytes, solid-state electrolytes appear to be a good alternative to conventional liquid electrolytes in methane electrochemical sensors.^163,164^Fig. 4B shows a solid-state methane sensor developed by Gross *et al.*^161^ In this configuration, Nafion was used as a solid-state electrolyte, which conducts the protons produced during the redox reaction of methane between the WE and the counter electrode.

#### Other types of methane sensors

Although most methane sensors are of the resistive and combustion types, other nanomaterial-enabled sensing modules have been reported for methane sensing. In the category of optical sensors, Mishra *et al.* utilized graphene-CNT/poly (methyl methacrylate) for SPR-based fiber optic sensing of methane.^165^ The shift in the resonance wavelength upon exposure to the gas was correlated to the methane concentration in the range of 10–100 ppm. Other optical sensing methods based on midinfrared light emitting diodes,^166^ refractive index-modulated optical fiber systems,^167^ photoacoustic spectroscopy,^168^ and photoelectrochemical detection^169^ have been demonstrated for methane quantification. In addition to optical sensing, methane detectors based on QCM^170^ and SAW^171^ have been developed, but their complexity in device design and user training has so far limited their applications in the mining sector.

### 4.2. Carbon dioxide sensors

#### Resistive gas sensors

**Fig. 5:**
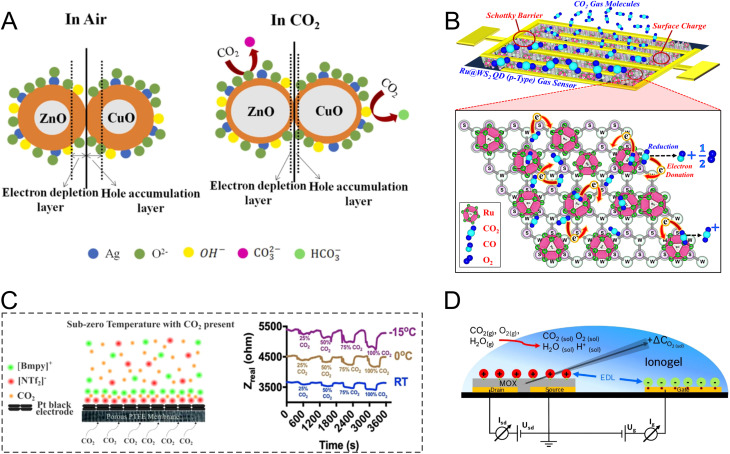
(A) Room-temperature CO_2_ sensing by a Ag-doped ZnO–CuO (p–n) heterojunction where the charge carrier movement provides more electrons for oxygen chemisorption. This is followed by a reduction in the HAL upon exposure to CO_2_.^178^ Reprinted with permission from ref. 178 Copyright 2023 Elsevier. (B) Illustration showing the CO_2_ sensing mechanism using Ru–WS_2_/Au electrodes.^187^ Adapted with permission from ref. 187 Copyright 2020 Institute of Physics. (C) EIS-based CO_2_ sensing *via* disruption of the IL assembly at the electrode interface due to CO_2_ inclusion.^192^ Reproduced with permission from ref. 192 Copyright 2023 American Chemical Society. (D) A FET sensor developed for CO_2_ sensing based on an electrolyte-gated mode using an IL and In_2_O_3_ as the electrolyte and channel forming layer, respectively.^203^ Reprinted with permission from ref. 203 Copyright 2020 Elsevier.

Similar to methane sensing, most of the resistive CO_2_ sensors are based on MOS materials. ZnO is one of the most popular MOS materials for CO_2_ sensing. ZnO is an n-type semiconductor and CO_2_ is an oxidizing gas. The chemisorption of CO_2_ on the surface of ZnO results in EDL widening and increased resistance. Structural doping,^172,173^ UV illumination,^174^ and heterojunction formation^175,176^ in a ZnO-based MOS have been studied to improve its CO_2_ sensing performance. In a study by Joshi *et al.*, a heterostructure of ZnO-calcium oxide (CaO) was shown to achieve sensitive (26–91%) and selective CO_2_ sensing at 150 °C in the range of 100–1000 ppm.^177^ The heterojunction was synthesized through chemical conversion of zinc hydroxide carbonate to ZnO by using calcium hydroxide, which enabled the formation of an n–n type nanointerface with extensive modulation of the potential barrier. The improved selectivity and sensitivity were attributed to higher CO_2_ adsorption on CaO due to the basicity of the Ca ion and improved charge-transfer reversibility. In another similar study, an Ag-doped ZnO–CuO heterojunction was utilized for room-temperature, sensitive CO_2_ detection within the range of 150–1000 ppm.^178^ The sensing mechanism, shown in Fig. 5A, was explained by the formation of a p–n heterojunction at the ZnO–CuO interface, which results in the movement of the electrons and holes (due to the difference in work functions of ZnO and CuO) and an increase in the number of free electrons near the surface. This is followed by chemisorption and ionization of oxygen and water molecules as well as a reduction in the HAL and an increase in the resistance of the sensor upon exposure to CO_2_. The dopant (Ag) improved the sensing performance due to the formation of a Schottky barrier, increased carrier mobility, and chemical sensitization.

Other sensing materials including SnO_2_,^179^ TiO_2_,^180^ CeO_2_,^181^ CuO,^182,183^ In_2_O_3_,^184^ rare-earth oxycarbonates and oxides,^185^ bismuth oxide (Bi_2_O_3_),^186^ and tungsten disulfide (WS_2_)^187^ have been explored for CO_2_ sensing. In a study by Zito *et al.*, yolk–shell CeO_2_ nanoparticles with high surface area and enhanced gas diffusion were explored for CO_2_ sensing.^181^ Their sensor showed fast response, stability, and high sensitivity to CO_2_ at 100 °C owing to the high adsorption capacity of the yolk–shell nanoparticles. In another study, quantum dots (QDs) of Ru-decorated WS_2_ were applied for room-temperature CO_2_ sensing within the concentration range of 500–5000 ppm.^187^ In the case of WS_2_, which is a p-type semiconductor, the sensing mechanism (Fig. 5B) was explained by CO_2_ chemisorption and breakage into CO and O_2_ leading to electron donation to the sensing interface, a decrease in the concentration of holes, and an increase in the resistance. Ru was assumed to have a catalytic role enabling fast CO_2_ reduction. Moreover, the presence of Ru led to a reduction in the ohmic loss and rectification at the interface of Ru–WS_2_/Au electrodes (an electrode on the sensor substrate).

In addition to conventional oxides based on single metallic elements, high-entropy metal oxide nanoparticles have been used for room-temperature and wide-range CO_2_ sensing (250–10,000 ppm). Gd_0.2_La_0.2_Y_0.2_Hf_0.2_Zr_0.2_O_2_ (Y-HEC) was explored for CO_2_ sensing using three different electrodes including Ag, Au, and indium tin oxide (ITO).^188^ The results show that Y-HEC made a perfect ohmic contact with the Ag electrode where the total resistance of the sensor was only controlled by the channel resistance of the sensing material without any contribution from the contact resistance at the metal–semiconductor interface. On the other side, a Schottky contact was formed at the Y-HEC/ITO and Y-HEC/Au interface with a lower Schottky barrier height (SBH) in the case of ITO. The highest response was obtained at the ITO interface, which revealed the vital role of the Schottky contact in gas sensor performance. Exposure to CO_2_ and release of electrons at the interface result in a downward shift in the level of the conduction band, which is followed by Schottky barrier modulation (SBM) and a reduction in the SBH. In gas sensors based on SBM, the presence of an optimized SBH leads to improved sensor performance. The lower sensitivity in Y-HEC/Au, compared to ITO one, was attributed to a large SBH, which prevented effective charge transfer.^188^

#### Electrochemical sensors

Electrochemical sensors based on potentiometric, amperometric, and impedance measurements have been employed for CO_2_ sensing. Among them, potentiometric sensors suffer from limited sensitivity as they respond to changes in EMF (electromotive force) against concentration on a logarithmic scale.^189^ Amperometric sensors are the most common module for electrochemical sensing of CO_2_ owing to their sensitivity, selectivity, ease of operation, and facile data interpretation.

Different types of solid and liquid electrolytes have been exploited for CO_2_ electrochemical sensing. IL-based CO_2_ sensors have been developed to take advantage of the tunable composition of ILs to enhance CO_2_ solubility and detection selectivity.^190^ In a study by Fapyane *et al.*, a mixture of IL 1-ethyl-3-methylimidazolium dicyanamide (EMIMDCA) and dimethylformamide (DMF) was used for the amperometric sensing of CO_2_*via* its reduction on the Ag WE.^191^ The addition of DMF reduced the response time and overpotential of CO_2_ reduction due to a decrease in stability of the CO_2_–EMIMDCA complex. The IL mixture enabled quantitative measurements of CO_2_ in the range of 0–4.62 kPa with a LOD of 0.5 kPa. In another study, Sridhar *et al.* developed an IL-based CO_2_ sensor operated based on the impedance readout.^192^ They investigated their gas sensing setup at different temperatures using a Pt black electrode. A decrease in the real component of the resistance was observed upon CO_2_ exposure, which was attributed to CO_2_ inclusion and disruption of the cation–anion interaction in molecularly structured IL film formed at the interface (Fig. 5C). At lower operating temperatures, increased viscosity of ILs and formation of a dense film made of ionic charges appear to increase the sensitivity of the sensor.

In addition to ILs, solid electrolytes are another group of popular electrolytic media widely employed for CO_2_ electrochemical sensing.^193^ Yttria-stabilized zirconia (YSZ) is often utilized as such an electrolyte, which has shown promise for amperometric measurements of CO_2_ up to the concentration level of 10% within the temperature range of 600–750 °C.^194^ In a study on the development of solid electrolytes for CO_2_ sensing, Ma *et al.* introduced Y-doped La_9.66_Si_5.3_B_0.7_O_26.14_ (Y-LSBO) as the electrolyte, which was coated with a working electrode film made of a Li_2_CeO_3_–Au–Li_2_CO_3_ composite.^195^ In this layered assembly, Li_2_CeO_3_ functions as an ionic bridge between the solid electrolyte with O^2−^ conductivity and Li_2_CO_3_ as the Li^+^ conductor. The sensor was operated based on the EMF readout and showed a Nernstian behavior for CO_2_ measurements within the range of 400–4000 ppm at 400 °C.

Apart from the reports replying only on the electrochemical input, a nanocomposite of ZnO/MoS_2_/reduced graphene oxide (rGO) was reported to allow sensitive photoelectrochemical sensing of CO_2_. In this case, a heterojunction was formed at the ZnO–MoS_2_ interface, and rGO functioned as a conducive bridge to facilitate the electron transfer. This assembly enabled CO_2_ detection in the range of 10–7820 ppm with a LOD of 10 ppm and response time of 10 s.^196^

#### Optical sensors

A variety of nanomaterial-incorporated optical CO_2_ sensors based on SPR,^197^ colorimetry,^198^ and infrared spectroscopy^199^ have been developed. In SPR-based CO_2_ sensors, CNTs are the most utilized plasmonic materials owing to their high affinity to CO_2_. However, poor selectivity and the existence of interfering excitation regions limit the application of CNT sensors in challenging mining environments.^200^

Colorimetric CO_2_ sensing is usually carried out by using semiconductor QD nanocrystals where a change in the emission intensity and/or blue or red shift can occur upon CO_2_ adsorption.^198,200^ In such sensors, although colorimetric measurements allow CO_2_ sensing in a cost-effective and facile manner, the semi-quantitative readout and poor long-term stability of QDs make them a less popular choice for the mining industry.

In CO_2_ sensors based on IR spectroscopy, nanomaterials are incorporated either into the light emitting source or the photodetector part.^200^ Such sensors normally have a chamber configuration, where certain IR wavelengths are absorbed by CO_2_ molecules. Having the CO_2_ IR spectrum as the readout, these sensors offer high accuracy, fast response, and durability. Their drawbacks include high cost, device complexity, and difficulty to scale up.

#### Other types of sensors

Based on our literature review, CO_2_ sensing is dominated by electrochemical and MOS-type resistive sensors. Other less common CO_2_ nanosensors rely on SAW,^201^ QCM,^202^ and FET.^203^ An advantageous version of such sensors with potential applications in mining, was introduced by Ersoez *et al.*^203^ Their sensor was based on a new concept of an electrolyte-gated transistor (EGT). As shown in Fig. 5D, they used an In_2_O_3_ film as a channel-forming layer in a FET and [EMIM][BF_4_] IL as an electrolyte separating the gate from the channel. Exposure to CO_2_ caused O_2_ depletion at the MOS interface resulting in an increased conductivity of the sensor. In this case, the IL provides a medium for dissolution of the gaseous reactants and modulates the charge carrier distribution in a MOS *via* the formation of an electrical double layer. This configuration enabled CO_2_ quantification in the range of 400–4000 ppm with a sensitivity of 0.1%/ppm and a recovery time of 20 s.

### 4.3. Carbon monoxide sensors

#### Resistive sensors

**Fig. 6:**
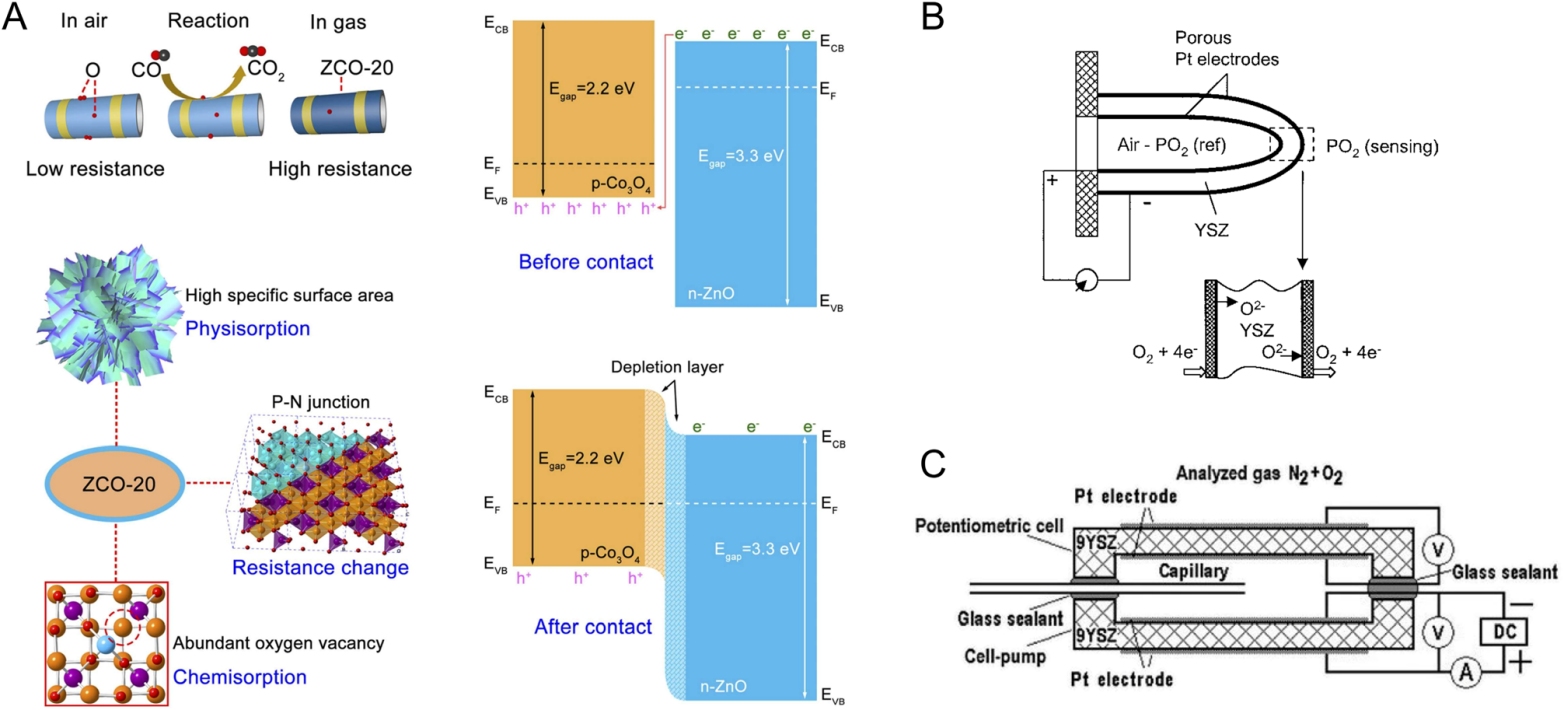
(A) Zeolitic imidazolate framework-derived n-ZnO/p-Co_3_O_4_ nanomaterials for CO sensing. The sensing nanomaterial presents many oxygen vacancies and enables strong chemisorption.^212^ Reprinted with permission from ref. 212 Copyright 2023 Elsevier. (B) Schematic of a potentiometric O_2_ sensor based on YSZ solid electrolyte.^79^ Adapted with permission from ref. 79 Copyright 2003 Springer. (C) A typical configuration of a mixed mode potentiometric–amperometric O_2_ sensor.^232^ Reprinted with permission from ref. 232 Copyright 2022, The Authors, under Creative Commons Attribution (CC-BY) license, published by Multidisciplinary Digital Publishing Institute.

Functional nanomaterials utilized in CO sensors include n-type^204^ and p-type^205^ MOSs as well as polymers.^206^ Considering CO as a reducing gas, CO interactions with an n-type MOS remove the ionized oxygen species, inject electrons back to the MOS, and decrease the overall electrical resistance of the sensor. A variety of nanostructures based on n-type MOSs of SnO, ZnO, In_2_O_3_, TiO_2_, WO_3_, and CeO_2_ have been employed for CO sensing.^207^ In a recent study, the CO sensing mechanism of a composite of SnO_2_/SiO_2_–PdO_*x*_ was studied in dry and humid air using DRIFT (diffuse reflectance infrared Fourier transform spectroscopy) analysis.^204^ The spectra revealed the contribution of PdO_*x*_ to CO oxidation and the role of SiO_2_ in preservation of the bridge oxygen atoms on SnO_2_ as well as the prevention of carbonate poisoning by decreasing the basicity of the sensing interface.^204^ In addition to n-type semiconductors, p-type MOSs have also been employed for CO sensing due to their higher catalytic activity and less temperature dependency of their conduction at elevated temperatures.^205,207^ CuO^205^ and Co_3_O_4_ ^208^ are among popular p-type MOSs for CO sensing. Regardless of the MOS type, doping,^209^ heterojunction assembly,^210^ and nanocomposite formation^211^ have been used to enhance CO sensing performance of MOS-based sensors. In a recent study by Yuan *et al.* porous nanoplates of n-ZnO/p-Co_3_O_4_ (Fig. 6A), derived from the zeolitic imidazolate framework, were used for selective and sensitive CO sensing.^212^ This nanomaterial exhibited a large surface area and high level of oxygen vacancy in the crystal structure, thus allowing strong chemisorption of CO molecules and high sensitivity with a response value of 35.4. According to the results, inclusion of Zn-based components appeared to be essential for anti-interference, *i.e.*, selectivity against interferents such as CH_4_, H_2_S, nitric oxide (NO), ammonia (NH_3_), and H_2_.

#### Electrochemical sensors

Electrochemistry is one of the best developed detection methods for CO sensing. Among different electrochemical modules, amperometry is the most utilized technique, where the current produced upon oxidation of CO to CO_2_ is tracked against time.^92^ In addition to amperometric sensors, there are a few demonstrations of potentiometric^213,214^ and EIS-based^215^ systems for CO sensing.

CO electrochemical sensor development mainly explores WE materials and electrolytic media. For the WE materials, metallic^216^ and metal oxide-based^217^ nanostructures as well as CNTs^218^ have been shown to provide effective sensing interfaces for CO electrochemical sensing. A Pt microdisk electrode modified with multi-walled CNTs (MWCNTs) was found to have a catalytic effect on CO oxidation with a reduced overpotential. The incorporation of MWCNTs allowed CO sensing within the range of 0.72–52 μg ml^−1^ with a LOD of 0.60 μg ml^−1^.^219^ Similarly, a nanocomposite of Pt–Ni alloy deposited on polyaniline-MWCNTs exhibited a bifunctional catalytic activity toward CO oxidation while neighboring Ni removed the reaction intermediates. A linear sensing response was obtained within the range of 1.0–50 μM with a LOD of 0.5 μM.^220^ The last two mentioned studies both used a liquid electrolyte of perchloric acid for sensing. To fabricate robust and durable sensors specifically suited for mining environments, electrolytic media other than aquatic solutions are required. To this end, electrochemical CO sensors based on solid electrolytes and ILs have been reported.^221,222^ Both inorganic and polymeric solid electrolytes^223^ have shown promises for CO sensing. In one of the earliest reports, a semipermeable and proton-conductive Nafion membrane was used to cover the surface of all three electrodes (sputtered Pt films served as the working and counter electrodes and a sputtered Au film was used as the reference electrode) required for electrochemical sensing.^223^ The sensor showed excellent durability of >2 year lifetime, a working range of 0–2000 ppm, and a response time of 30 s for CO sensing. The remarkable sensing performance was attributed to CO permeability of Nafion and the higher oxidation rate of CO. In a study of inorganic solid electrolyte-based CO sensors, Phawachalotorn *et al.* used a Fe-doped La_0.8_Sr_0.2_GaO_3_ solid electrolyte in combination with electrocatalysts Au 10 wt%–In_1.9_Sn_0.1_O_3_ (ITO955) and RuO_2_–La_0.6_Sr_0.4_CoO_3_ (LSC64) for amperometric CO sensing.^224^ This type of sensor operated within a temperature range of 300–500 °C and showed a sensitive (8.83 mA per decade) and selective (over CH_4_, CO_2_, and H_2_) response to CO.

#### Other types of sensors

In addition to the traditional CO sensors developed for mining applications such as the chemiresistive and electrochemical types, other less developed CO sensors include QCM,^225^ FET,^226^ SAW,^227^ and optical (*e.g.,* SPR,^228^ reflectometry,^229^ and fluorescence,^230^) sensors. In particular, FET-based CO sensors are promising for field applications. Singh *et al.* demonstrated room-temperature CO sensing by using Zn-doped In_2_O_3_ nanowires (NWs) in an FET configuration.^231^ Zn doping enhanced the sensor response and enabled CO sensing within the range of 1–5 ppm with a selective response over NO and nitrogen dioxide (NO_2_).

### 4.4. Oxygen sensors

#### Electrochemical sensors

Electrochemical O_2_ sensors based on solid electrolytes are the most studied commercial sensors for O_2_ measurements in the gas phase. Traditionally, O_2_ sensing is carried out at high temperature in a planar configuration using YSZ as a solid electrolyte with oxygen conduction. As can be seen in Fig. 6B, the sensor is constructed in a multilayered configuration with two Pt electrodes exposed to the test gas stream and the reference gas. The electromotive force, resulting from the oxygen pressure disparity between the two Pt electrodes, serves as the measured signal readout. Upon exposure to the target gas stream, the molecular oxygen adsorbs on the Pt sites, which is followed by O_2_ dissociation to atomic oxygen and its ionization/reduction at the electrode–electrolyte-gas boundary, referred to as the triple phase boundary (TPB). As YSZ has a high level of oxygen conduction, the chemical potential of reduced oxygen species is not changed in the solid electrolyte media. Thus, the difference in chemical potential of O_2_ exists at the test stream, and the reference stream generates an EMF for potentiometric O_2_ sensing.^79,91^ Advances in such O_2_ sensors involve the replacement of the reference Pt/gas interface, with metal/metal oxide interfaces to simplify the sensor configuration and improve its applicability. Several metal/metal oxide interfaces based on Sn, In, Ni, and Ru have been explored as the reference, and they can maintain a desirable oxygen partial pressure at a given temperature (<500 °C).^232^

Apart from commercial potentiometric oxygen sensors, amperometric modules have been employed to remove the logarithmic dependency of concentration to the readout and allow O_2_ sensing within a wider concentration window. The amperometric sensors record the current generated from oxygen reduction and have been used for sensing of O_2_ dissolved in liquid electrolytes^233^ and O_2_ in the gas phase at the interface of a solid electrolyte and an electrode.^232^ In the case of solid electrolytes, in addition to the conventional YSZ,^79^ samarium (Sm)-doped CeO_2_ has high oxygen ion conductivity enabling amperometric O_2_ sensing within the range of 100–500 ppm at 550 °C.^234^

The limiting current in these sensors depends on the applied direct current (DC) potential utilized to pump molecular oxygen to the working electrode surface. Depending on the target test stream, different potentials may be required to achieve a steady-state current. To compensate for this dependency and improve the reliability of O_2_ amperometric sensors, a combined amperometric–potentiometric sensing technique has been introduced. Fig. 6C shows a typical design consisting of two electrochemical chambers of an amperometric and a potentiometric cell, respectively. In the amperometric chamber, O_2_ is pumped and measured based on the limiting current, and the potentiometric chamber records the EMF value and provides additional information on sensor performance and the analyte concentration.^79,232^

#### Resistive sensors

O_2_ resistive sensors usually operate based on O_2_ chemisorption on MOS materials. Among a variety of MOS-based O_2_ sensors, Ti-, Ga-, and Ce-based semiconductors are the most common. Semiconductors of TiO_2_, SrTiO_3_, Ga_2_O_3_, CeO_2_, and Nb_2_O_5_ with n-type characteristics have shown sensitivity toward O_2_ molecules.^79,235^ The sensitivity is obtained through a sequence of events including the formation of oxygen adsorbents, occupation of oxygen vacancies in the n-type MOS, a reduction in the concentration of electrons as charge carriers, and an increase in the resistance of the sensor. So far, several O_2_ resistive sensors based on TiO_2_ thick films have made it into the market.^235^ New advances have focused on the exploitation of nanostructures and thin films of TiO_2_ (either in a pristine or composite form) to further enhance O_2_ sensing properties.^236^ Thin films of TiO_2_ with a particle size of ∼34 nm were found to have a high response to O_2_ at low operating temperatures within 150–300 °C.^237^ Strontium titanate (SrTiO_3_) is another popular Ti-based semiconductor with a perovskite structure widely employed for high temperature O_2_ sensing. At a low oxygen partial pressure, oxygen vacancies in SrTiO_3_ result in an n-type behavior while at a high oxygen partial pressure (>1 Pa), Sr vacancies dominate the semiconductor structure giving rise to a p-type behavior of the sensing material.^79^ In this case, doping the structure with donor or acceptor elements (*i.e.,* La or Fe, respectively) brings about a shift in the p–n transition and a monotonic signal change *versus* the O_2_ concentration in the donor-doped structures.^238^

Among the other n-type MOS interfaces reported, CeO_2_ is a well-researched material for O_2_ sensing.^239^ The Ce atoms inside the CeO_2_ crystalline lattice possess variable oxidation states of Ce^3+^/Ce^4+^, which leads to oxygen storage capability and fast oxygen vacancy diffusion in CeO_2_. The latter feature was attributed to the significant reduction in the response time of the O_2_ sensor.^79,235^ Films of CeO_2_ have been reported to have response times within 5–10 ms.^197^ Moreover, the addition of Zr to Ce forms a mixed oxide phase and further increases the charge carrier mobility resulting in a response time within the range of 1–20 ms.^240^

Several p-type semiconductor interfaces exhibiting temperature-independent resistivity have also been explored for O_2_ sensing. Examples of such temperature-independent interfaces include lanthanum cuprate (La_2_CuO_4+*δ*_), SrTi_1−*x*_Fe_*x*_O_3_ (STF), and BaFe_1−*y*_Ta_*y*_O_3_.^235^ The temperature independency of resistance in STF was explained by the compensation of temperature-induced formation of charge carriers due to strong temperature-dependent mobility of holes and a decrease in the bandgap upon the incorporation of Fe electronic bands into SrTiO_3_.^241^

#### Other types of sensors

Other types of O_2_ sensors utilizing nanomaterials, less suitable for harsh mining environments, include FET^242^ and photoluminescence sensors.^243^ Fan *et al.* incorporated single-crystal ZnO NWs into the FET configuration for O_2_ sensing,^244^ and reported thinner NWs to exhibit higher sensitivity enabling O_2_ measurements in the range up to 50 ppm.

An amine-functionalized silver-chalcogenolate-cluster-based MOF was employed as a dual fluorescence-phosphorescence probe for O_2_ sensing. Ratiometric sensing was conceived as O_2_-induced phosphorescence quenching relative to an O_2_-independent fluorescence signal as the readout. It was shown that a second functionalization with methyl moieties can interfere with the quenching process providing a wider sensing concentration range of 0.5–20 ppm. The sensor displayed a response time of 0.3 s.^245^

## 5. Direct electrodeposition of charge-transfer complex-based nanosensors

**Fig. 7:**
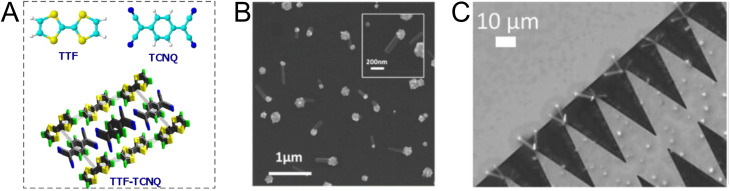
(A) Schematic illustration of molecular packing in TTF-TCNQ CTC.^254^ Adapted with permission from ref. 254 Copyright 2009 Institute of Physics. (B) Seed-mediated controlled growth of potassium tetracyanoplatinate sesquihydrate nanowires on gold nanoparticles.^251^ Reprinted with permission from ref. 251 Copyright 2017 Taylor & Francis. (C) Substrate-directed electrocrystallization of tetrathiafulvalene bromide (TTFBr) nanowires on patterned gold electrodes.^252^ Reprinted with permission from ref. 252 Copyright 2023 John Wiley & Sons, Inc.

Charge-transfer complexes (CTCs) refer to a group of organic and organometallic conductors and semiconductors with unique electrocrystallization properties. CTC molecular assemblies are made through a charge transfer process between electron acceptor and electron donor counterparts giving rise to conductive to semiconductive characteristics.^246–248^Fig. 7A shows the molecular packing structure in tetrathiafulvalene-7,7,8,8-tetracyanoquinodimethane (TTF-TCNQ), one of the most studied CTCs.^249^ In such a molecular assembly, electrons are delocalized along the stacks of electron acceptor/donor molecules, and conductive crystals are grown along the *c*-axis leading to a 1D columnar structure.^250^ These 1D semiconducting crystals offer numerous synthetic chemistry variations for scalable manufacturing of gas nanosensors. We have previously shown the possibility of controlled electrocrystallization of 1D CTCs through seed mediation^251^ (Fig. 7B) and substrate patterning^252^ (Fig. 7C). These strategies offer a versatile approach for precise electrodeposition of CTC nanosensors directly on sensor circuitry. Direct electrocrystallization of such nanowire sensors will enable low-cost production of high-quality crystalline nanomaterials as an effective additive manufacturing strategy to overcome a major challenge in nanosensor commercialization.^253^

CTC electrocrystallization has shown promises to create gas sensitive interfaces owing to CTC's semiconducting characteristics and tunable chemistry. Various compositions of CTCs can be exploited using electrochemistry (Table 2). Variations in molecular stacking, counterions, or stoichiometry of CTCs lead to different physical and chemical properties, which can be adjusted to achieve selective interactions with target gas molecules.^255^ There are several literature reports on CTCs being used for detecting both reducing^256^ and oxidizing^257^ gases. Our group has demonstrated the sensing performance of tetrathiafulvalene bromide (TTFBr_0.76_) nanowires electrodeposited directly on patterned electrodes for ammonia measurements.^256^ According to our observations, depending on the CTC stoichiometry, different sensor readouts of ammonia were accomplished. In insulating TTBr_1.0_, exposure to reducing ammonia increases the concentration of charge carriers and reduces the resistance. In the case of conductive TTFBr_0.76_, electron injection neutralizes TTF^+^ and obscures intermolecular donor–acceptor interactions resulting in an increased resistance.^256^ In another research study on gas sensing capability of CTCs, Wang *et al.* applied the TTF-TCNQ complex for measuring and differentiating alkyl amines and aromatic amines.^258^ In both cases, after exposure to the amines, the electrical current readout over TTF-TCNQ decreased due to donor–acceptor interactions between amines and TCNQ, which competes with that of TTF-TCNQ. Given the higher basicity of alkylamines than that of aromatic amines, they form a stronger bond with TCNQ, and an irreversible signal was observed in this case, while in the case of aromatic amines, the sensor signal was recovered after a few seconds. Hence, the recovery behaviour of the sensor was used as a criterion for distinguishing between alkyl amines and aromatic ones.^258^ Another research study has demonstrated the sensitivity of the TTF-TCNQ complex to oxidizing gas species, such as CO_2_, O_2_, and NO_2_, through alteration of the charge transfer in the complex resulting in a reduction in conduction.^259^

**Table 2:** Donor and acceptor counterparts commonly used for the synthesis of CTCs

Common donor components	Common acceptor components
Tetrathiafulvalene (TTF)	7,7,8,8-Tetracyanoquinodimethane (TCNQ)
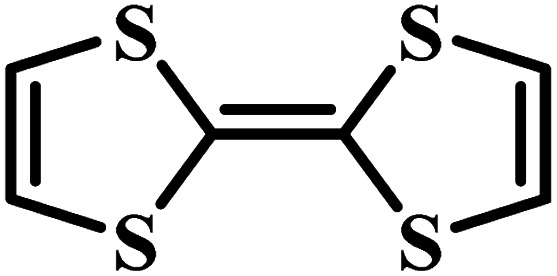	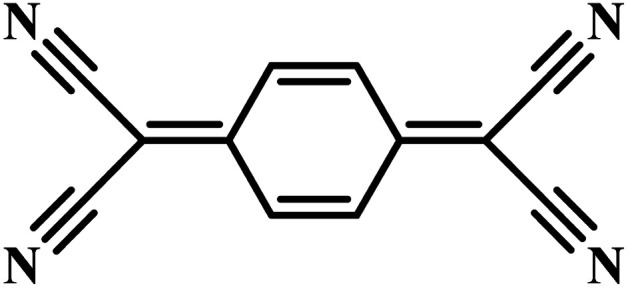
Bis(ethylenedithio)tetrathiafulvalene (ET)	Cl^−^, Br^−^, I^−^, PF_6_^−^
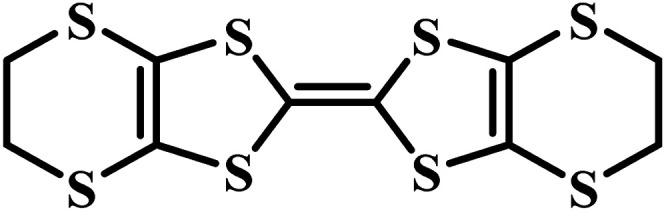	
Tetramethyltetraselenafulvalene (TMTSF)	
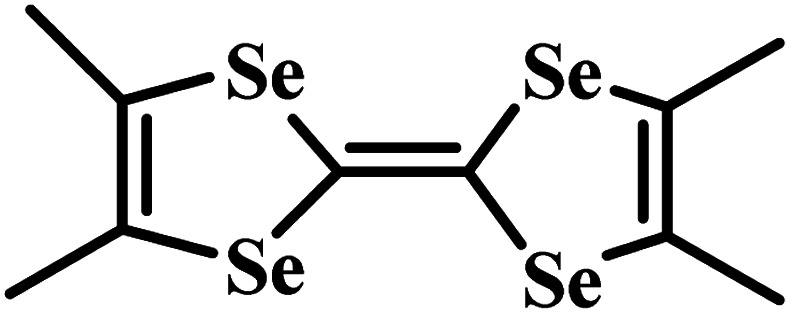	
Ag, Cu, and Co	

Taken all together, CTCs present a potential application for gas monitoring in mines. Compared to MOS-based systems, the sensing interfaces based on CTCs offer a versatile manufacturing technology, possess tailorable chemistry, and operate at room temperature. CTC nanosensors could respond to reducing and oxidizing gas molecules of CH_4_ and CO, respectively, as demonstrated in a limited number of studies on similar molecules so far.^258,259^ The possibility of fabricating aligned nanowires through a simple substrate-directed electrochemical route offers a means for constructing nanosensor arrays capable of multiplexed measurements in a single wearable or portable and networkable device.

## 6. Conclusions and future remarks

The present review provides an overview of the various sensing methodologies and nanomaterials employed for the detection of critical gases of interest in the mining industry. The sensing mechanisms and the interactions between the target gas molecules and the sensing nanomaterial are discussed for each sensing module and gas type. Based on our review of literature, it is evident that the emergence and extensive application of nanomaterials have advanced the gas sensor field rapidly and significantly. So far, a large variety of nanomaterials with certain characteristics, depending on the sensing method, have been incorporated into gas sensors for different gases. However, despite the broad development of a diverse array of nanomaterials, new understanding of the interactions between the adsorbed gas and the sensing interface has been lacking in the literature. In this regard, most literature reports rely on generally accepted mechanisms and theories for describing their sensing systems. Mechanistic investigations through actual experiments to provide deeper understanding of the sensing pathways and the role of each component in a typical composite material have been lacking. In addition to this, the majority of gas sensors developed to date (with the exception of certain optical and electrochemical systems) exhibit cross-sensitivity to both the target gas and background gases. Selectivity in gas sensors remains to be improved. To complement the development of nanomaterials with inherent gas selectivity, it is essential to incorporate surface functionalization, external filtering, and various data optimization methods to achieve the ultimate selectivity, enabling applications in highly variable mining environments.

The integration of nanostructures into gas sensing interfaces holds great promise for fabricating miniaturized, efficient, and accurate portable and wearable devices. Nanoscale structures provide a larger responsive interface over a given sensor area, leading to faster response times and higher sensitivity. The utilization of these portable devices enables early warnings over a wide area, making them easily networkable and applicable in the mining sector.

While the incorporation of nanomaterials into gas sensing platforms has greatly improved their applicability and performance, there still remain challenges in terms of developing facile, cost-effective, reproducible, and scalable fabrication techniques for integrating nanostructures into sensing interfaces. The direct electrocrystallization of nanostructures on patterned substrates, as discussed in the preceding section, offers a promising approach to overcome challenges in the integration of nanomaterials into sensing interfaces. Efforts aimed at upscaling laboratory nanosensor production, guided by new science and engineering principles, are crucial to propel scientific discoveries towards commercialization and widespread industry adoption.

## Conflicts of interest

There are no conflicts to declare.

## Supplementary Material
